# Supplementary material for: Youth-friendly health service in Ethiopia: Assessment of care friendliness and user’s satisfaction
Source: PLoS One. 2024 Jul 16;19(7):e0307142. doi: 10.1371/journal.pone.0307142 (PMC11251592; doi:10.1371/journal.pone.0307142)
Supplement: S1 File — (PDF) [file pone.0307142.s001.pdf]

S1 File. English version questionnaire

This questionnaire is prepared to examine the degree of friendliness with adolescent and youth-friendly health services and Adolescents' and Youths' SRH exposure, and their satisfaction with the services in Addis Ababa city administration and the East Shewa zone in the Oromia region, Ethiopia

Section I. Identification

1.1. Region________________
1.2. Zone ________________
1.3. Woreda_________________
1.4. Town ________________
1.5. Kebele_______________
1.6. Name of the Health Facility __________________
1.7. Date of Interview _________________________
1.8. Questionnaire code
1.9. Name (code) of the interviewer

Section II. Background information

1. Sex: 1. Male 2. Female
2. How old were you on your last birthday? **[Enter number]** ______
   1.____________ Years 2. Don't know/No answer
3. What is your Religion?
   1. Orthodox 2. Muslim 3. Catholic 4. Protestant 5. Other, (specify)____
4. What is your Marital Status?
   1. Single 2. Married 3. Divorced/separated 4. Widowed 5. Other, (specify)____
5. If ever married, at what age did you first get married? **[Enter number]**
   _____ Years
6. What is the highest level of school you have completed?
   1. Primary level (1-8) 2. Secondary level (9-12) 3. Over secondary level 4. Other, (specify)____
7. What is your Current educational status?
   1. Currently in school 2. Out-of-school

Section III. Sexuality, pregnancy, and contraception

8. Have you ever had sexual intercourse?
   1. Yes 2. No **[Skip to Q. 13]** 99. Don't know/No answer **[Skip to Q. 13]**
9. If yes, at what age did you first have sexual intercourse? **[Enter number]**
   1. _______years. 99. Don't remember/No response
9. With how many partners have you had sexual intercourse? **[Enter number]**
   1. ______ partner(s). 2. Don't remember/No response
10. During the last six months, have you had sexual intercourse?
   1. Yes 2. No **[Skip to Q. 21**

11. If yes, did you use a method to prevent pregnancy and diseases?

1. Yes 2. No **[Skip to Q. 15]** 3. Don't remember/No response **[Skip to Q. 15]**

12. Which methods did you usually use to prevent pregnancy or diseases in the last six months? **(Multiple responses are allowed)**

1. Condom 2. Pill 3. Injectables 4. IUD/Loop 5. Norplant 6. Safe Days/ Rhythm
7. Withdrawal before ejaculation 8. Other, (specify)____

13. Which contraceptive method is used by a woman who has had unprotected sex or raped within 72 hrs to prevent pregnancy?

1. Condom 2. Injectable 3. Emergency contraceptive 4. Norplant 5. Don't know/No response ______

14. During which part of the monthly cycle does a woman have the greatest chance of becoming pregnant?

1. During her period 2. In the middle of her cycle 3. Right after her period has ended
4. Just before her period begins 5. Any time 6. Don't know/No answer

For Sexually Active Female Respondents

15. Have you ever been pregnant?

1. Yes 2. No 3. Don't know/No answer

16. If yes, what was your age when you were first pregnant? **[Enter number]**

1. _____ years 2. Don't remember/No response

17. How did the pregnancy end?

1. I gave birth 2. I had still birth 3. I had spontaneous abortion 4. I had induced abortion

18. if you had induced abortion where did you terminate the pregnancy?

1. At the project health facility 2. At private clinic 3. At abortionist's house
4. Induced it myself using different drugs 5. Other specify________

19. Do you use contraceptives?

1. Yes 2. No 99. Don't know/No answer

20. If yes from where do you collect the contraceptive?

1. From the project health facility 2. From private clinic 3. From pharmacy
4. Other, (specify)____

Section IV. Exposure to AYFH program/service

29. Do you have the information that there is AYFH service in _________________health facility?

1. Yes 2. No

30. If yes, from whom did you get the information?

1. Friends 2. Health personnel 3. Teachers 4. Parents 5. Public announcement
6. Other, (specify)____

31. What services are provided in the health facility for the youth? **(Multiple responses are allowed)**

1. Comprehensive Abortion Care (CAC) 2. Comprehensive Contraceptive (CC)
3. Pregnancy Test 4. STIs Diagnosis and Treatment 5. Other, (specify)____

32. Have you ever visited the AYFH service in the health facility?

1. Yes 2. No

33. What was the reason for your visit? **(Multiple responses are allowed)**

1. For Comprehensive Abortion Care (CAC) 2. For Contraceptive 3. For pregnancy test 4. For STIs diagnosis and treatment 5. Other, (specify)____

34. Are there obstacles for youth to getting AYFH services from the health facility?

1. Yes 2. No

35. If yes, what are the obstacles? **(Multiple responses are allowed)**

1. The health facility is too far 2. Fear not to be seen by family members/neighbours 3. Due to lack of privacy and confidentiality 4. Due to providers' judgmental attitude 5. Waiting time is too long 6. Working hours are not convenient 7. Have no separate place/room for the youth 8. Other, (specify)____9. Don't know/No response

36. How should you think the AYFH service be arranged for the youth in the health facility? **(Multiple responses are allowed)**

1. Arrange a special place/room for the AYFH services 2. Organize special time for the AYFH service on the working days
3. Plan the AYFH service to be provided on weekends 4. Arrange a reading corner in the health facility 5. Have recreational facilities 6. Other, (specify)____

37. Whom would you prefer as an AYFH service provider for the youth? **(Multiple responses are allowed)**

1. Young provider of the same sex 2. Young provider of any sex 3. Adult providers of the same sex 4. Adult providers of any sex 5. Any provider 6. Other, (specify)_____

Section V. AYFH services friendliness assessment tool

This tool is prepared to examine the level of care-friendliness with AYFH services from users' perspective. The questions are designed to align with WHO's YFHS Quality of Care Standards.

S.n.	Questions	Yes/No/NA	Comments
**1.**	**Equitable**		
1.1.	Are procedures in place to ensure that no young people are excluded from services?		
**2.**	**Accessible**		
2.1.	Is information and referrals provided about where young people can access other youth-friendly health or social services in the community?		
2.2.	Have you found the waiting times too long before seeing the health-care providers?		
2.3.	Are the working days and working hours of the health facility convenient for you?		
2.4.	Are services located in an area that is accessible to youth and safe for them to travel to?		
2.5.	Are services free of cost or affordable for young people?		
2.6.	Are you able to access all of their health services in one visit?		

2.7.	Are there separate clinic hours or waiting areas just for young people?		
**3.**	**Appropriate**		
3.1.	Does the site have posters, brochures and other IEC materials that target young people, including information about their rights?		
3.2.	Are the services advertised to young people in places where they congregate (e.g., schools, youth clubs, recreation centers, etc.)?		
3.3.	Is peer support or mentoring available?		
3.4.	Are educational activities youth-friendly and address topics of interest to youth? (e.g., role plays, theatre, games, etc.)		
4	**Acceptable**		
4.1.	Are youth involved in program design, delivery, and evaluation?		
4.2.	Are young people greeted warmly upon entering?		
4.3.	Are sessions conducted in an area that provides privacy so that nobody can see or hear the conversations taking place?		
4.4.	Do you think that your parents/guardians would be supportive of you coming to this health facility for reproductive health services?		
4.5.	Do you believe that the information you shared with the health-care provider will be kept confidential?		
5	**Effective**		
5.1.	Are condoms/other methods available to young people on-site?		
5.2.	Competent health-care providers		
5.3.	Does the site have a youth-friendly strategy or action plan in place?		
	**Other**		

Section VI. Adolescent and Youth Client Satisfaction Assessment tool

This tool is prepared to assess the level of client satisfaction with the service provided at adolescent and youth units

S. n	Questions	Yes/No/NA	Comments
1.	Was it easy for you to get to this healthcare site today?		
2.	Were the services that you came for affordable?		
3.	Did you find the waiting time acceptable?		

4.	Did the health provider tell you that everything you discuss would remain confidential?		
5.	Was anybody else in the room during your visit? If so, did the provider explain who they were?		
6.	Do you believe that others could hear your discussions with the health care provider when you were in the treatment/ consultation room?		
7.	Did the health care provider give you his/her full attention?		
8.	Did the health care provider seem interested in what you had to say?		
9.	Did the health care provider respect your opinion and decisions even if they were different from his/hers?		
10	Did you find the environment at the health service site welcoming?		
11.	Did you feel comfortable talking to all of the people working at the health facility?		
12.	Did you feel that you could talk about everything that you wanted to and ask all of the questions that you wanted to with your provider?		
13.	Did the health care provider explain everything that he or she was doing and about any services being provided?		
14.	Did you understand everything that the provider told you?		
15.	Did the health care provider refer you to any services that are not provided here?		
16.	Would you recommend this health site to a friend who needed similar help?		
17.	Is there a staff member who you feel works especially well with you? (*Describe what that person did to make you feel comfortable.*)		
18.	Based on your experience, what kinds of things would make you want to come back to this service site?		
19.	What kinds of things would discourage you from coming back?		
20.	Other		
