# Supplementary material for: Youth-friendly health service in Ethiopia: Assessment of care friendliness and user’s satisfaction
Source: PLoS One. 2024 Jul 16;19(7):e0307142. doi: 10.1371/journal.pone.0307142 (PMC11251592; doi:10.1371/journal.pone.0307142)
Supplement: S1 Table — (PDF) [file pone.0307142.s002.pdf]

S1. Table. Measurement/scoring sheet/ for the five domains of care friendliness

Domain	Characteristics/Questions	Response	Score
Equitable	Are procedures in place to ensure that no young people are excluded from services?	0=No, 1=Yes	Total "Yes" divided by total number of adolescent/youth clients interviewed
Absolute total score		0-1	Total score
Relative score (equity)		0 to 100%	$\left(\frac{Total\ score}{1}\right) * 100$
Accessible	Is information and referrals provided about where young people can access other youth-friendly health or social services in the community?	0=No, 1=Yes	Total "Yes" divided by total number of adolescent/youth clients interviewed
	Have you found the waiting times too long before seeing the health-care providers?	0=Yes, 1=No	Total "No" divided by total number of adolescent/youth clients interviewed
	Are the working days and working hours of the health facility convenient for you?	0=No, 1=Yes	Total "Yes" divided by total number of adolescent/youth clients interviewed
	Are services located in an area that is accessible to youth and safe for them to travel to?	0=No, 1=Yes	Total "Yes" divided by total number of adolescent/youth clients interviewed
	Are services free of cost or affordable for young people?	0=No, 1=Yes	Total "Yes" divided by total number of adolescent/youth clients interviewed
	Are you able to access all of their health services in one visit?	0=No, 1=Yes	Total "Yes" divided by total number of adolescent/youth clients interviewed
	Are there separate clinic hours or waiting areas just for young people?	0=No, 1=Yes	Total "Yes" divided by total number of adolescent/youth clients interviewed
Absolute total score		0-7	Total score
Relative score (accessibility)		0 to 100%	$\left(\frac{Total\ score}{7}\right) * 100$
Appropriate	Does the site have posters, brochures and other IEC materials that target young	0=No, 1=Yes	Total "Yes" divided by total number of

	people, including information about their rights?		adolescent/youth clients interviewed
	Are the services advertised to young people in places where they congregate (e.g., schools, youth clubs, recreation centers, etc.)?	0=No, 1=Yes	Total "Yes" divided by total number of adolescent/youth clients interviewed
	Is peer support or mentoring available?	0=No, 1=Yes	Total "Yes" divided by total number of adolescent/youth clients interviewed
	Are educational activities youth-friendly and address topics of interest to youth? (e.g., role plays, theatre, games, etc.)	0=No, 1=Yes	Total "Yes" divided by total number of adolescent/youth clients interviewed
Absolute total score		0-4	Total score
Relative score (appropriateness)		0 to 100%	$\left(\frac{Total\ score}{4}\right) * 100$
Acceptable	Are youth involved in program design, delivery, and evaluation?	0=No, 1=Yes	Total "Yes" divided by total number of adolescent/youth clients interviewed
	Are young people greeted warmly upon entering?	0=No, 1=Yes	Total "Yes" divided by total number of adolescent/youth clients interviewed
	Are sessions conducted in an area that provides privacy so that nobody can see or hear the conversations taking place?	0=No, 1=Yes	Total "Yes" divided by total number of adolescent/youth clients interviewed
	Do you think that your parents/guardians would be supportive of you coming to this health facility for reproductive health services?	0=No, 1=Yes	Total "Yes" divided by total number of adolescent/youth clients interviewed
	Do you believe that the information you shared with the health-care provider will be kept confidential?	0=No, 1=Yes	Total "Yes" divided by total number of adolescent/youth clients interviewed
Absolute total score		0-5	Total score
Relative score (acceptability)		0 to 100%	$\left(\frac{Total\ score}{5}\right) * 100$
Effective	Are condoms/other methods available to young people on-site?	0=No, 1=Yes	Total "Yes" divided by total number of

			adolescent/youth clients interviewed
	Competent health-care providers	0=No, 1=Yes	Total “Yes” divided by total number of adolescent/youth clients interviewed
	Does the site have a youth-friendly strategy or action plan in place?	0=No, 1=Yes	Total “Yes” divided by total number of adolescent/youth clients interviewed
Absolute total score		0-3	Total score
Relative score (effectiveness)		0 to 100%	$\left(\frac{Total\ score}{3}\right) * 100$
Global score /Friendliness	$\left(\frac{Total\ score\ for\ all\ domains}{total\ \#\ questions\ (20)}\right) * 100$		
