# Supplementary material for: Youth-friendly health service in Ethiopia: Assessment of care friendliness and user’s satisfaction
Source: PLoS One. 2024 Jul 16;19(7):e0307142. doi: 10.1371/journal.pone.0307142 (PMC11251592; doi:10.1371/journal.pone.0307142)
Supplement: Attachment — Submitted filename: Response to Reviewers.docx [file pone.0307142.s003.docx]

# Rebuttal Letter

Academic Editor

PLOS ONE

June 1, 2024

Dear academic editor,

Thank you for inviting us to submit a revised draft of our manuscript entitled, "Youth-friendly health service in Ethiopia: Assessment of care friendliness and user’s satisfaction" to PLOS ONE. We also value the time and energy you and the other reviewers invested in providing thoughtful feedback on how to strengthen our article. We are therefore delighted to resubmit our article for further consideration. We have made the required modifications in light of your extensive suggestions. We hope our edits and responses below satisfactorily address all the issues and concerns you and the reviewers have noted.

To facilitate your review of our revisions, the following is a point-by-point response to the questions and comments in a letter dated June 1, 2024

**ACADEMIC EDITOR’S COMMENTS**

First, thank you for your precious time, insightful comments, and suggestions for improving our paper.

1. **Academic editor’s comment:** Provide adequate justification for how the study areas are selected.

- **Response:** We value the time you took to fairly evaluate our methods section and point out any gaps. The study setting has been modified and the reason for selecting Addis Ababa city administration and East Shewa zone has been included in the document. The main reasons for selecting the study setting were, of course, the presence of functional youth-friendly health service corners in the health facilities and accessibility of the health facilities.

2. **Academic editor’s comment:** To make it more informative and ensure replicability, reorganize methods.

- **Response:** Thank you for suggesting a way to reorganize the methods section. We reorganized the methods as follows:
  - Study design, setting, and period
  - Study participants and selection procedure (inclusion/exclusion criteria are incorporated)
  - Sample size and sampling techniques
  - Data collection tools and procedures (the contents of the tool are described, the methods of data collection, data collectors and supervisors, and their qualifications are also included, the English version of the questionnaire is uploaded as supplementary material)
  - Study variables (the measurement of the five domains of care-friendliness including equity, accessibility, acceptability, appropriateness, and effectiveness prepared in a separate document and uploaded as supplementary material (to manage the space in the methods part)).
  - Data quality control
  - Data processing and analysis
  - Ethical consideration

3. **Academic editor’s comment:** Figures should be self-explanatory (add axis titles); describe all abbreviations used within tables as table footnotes

- **Response:** We have revised the title of our figures by adding the axis titles. Additionally, a description and a footnote are included for each abbreviation used within the tables

4. **Academic editor’s comment:** Discuss both the strengths and limitations of the study

- **Response:** Thank you for this suggestion. We have made revisions in both parts.

5. **Academic editor’s comment:** Include the following items (a rebuttal letter, a marked-up copy of your manuscript, and an unmarked version of your revised paper) when submitting your revised manuscript

- **Response:** Thank you for your suggestion. We have prepared all three documents as per the request and uploaded them as separate files

**REVIEWER 1 COMMENTS:**

We sincerely appreciate you taking the time to read our paper, giving us insightful feedback, and providing all useful comments.

6. **Reviewer 1 comment:** Unnecessary use of words like “we” in page 2 line 31-32. Revise it.

- **Response:** Thank you for your fair assessment. We have revised this part accordingly.

7. **Reviewer 1 comment:** What was the research gap regarding the intention of women to give birth at a health facility? If any put your strong justification in the final paragraph of the background.?????

- **Response:** Thank you for raising this question. We have modified the background section indicating the gap in evidence about our study. However, we believe that the intention of women to give birth at the facility does not directly align with our main research question.

8. **Reviewer 1 comment:** In the background part, you should describe YFS and YFS satisfaction briefly.

- **Response:** Thank you for this suggestion. We have explained AYFHS and Satisfaction with AYFH services in the background section.

9. **Reviewer 1 comment:** The problem was not well stated in the introduction part. What was the problem of friendliness? What was the problem of satisfaction? Consequences of poor satisfaction?

- **Response:** Thank you for making a fair assessment of this section. In addition to explaining the AYFH services, satisfaction, and friendliness of services, we have indicated the existing gaps in friendliness, satisfaction, and the consequences of low satisfaction among adolescents and youths with AYFH services. In short, we have stated the problem in a separate paragraph under the background section.

10. **Reviewer 1 comment:** Describe the characteristics of the study population regarding youth-friendly services and health facility availability/accessibility, trained health care provider availability, and other important information in your study setting.

- **Response:** Thank you for this suggestion. We have further modified the study setting, and now the study area's administrative layer, the overall population, the total adolescent population, and the accessibility of functional health facilities that provide services to youths and adolescents are all explained.

11. **Reviewer 1 comment:** I’m not satisfied with the selection criteria for selecting one health center from the east Shewa zone. Can we say it is representative of all other health facilities in the zone?

- **Response:** This is an interesting query. Modifications in the methods part particularly the study setting is made. We also believe that a single study site in East Shew cannot represent all the health facilities in the area. The rationale/reason for selecting the east Shewa zone of the Oromia region is explained.

12. **Reviewer 1 comment**: The study period must be specified. The specific date of commencement and final date of data collection was not specified. You said from August to October 2022. See page 6 line 100

- **Response:** Thank you for making a fair assessment of this part. The study was conducted from August 1 to October 28, 2022, and this is presented in the study period part of the paper.

13. **Reviewer 1 comment**: Measurement of accessibility, equity, acceptability, appropriateness, and effectiveness must be clearly mentioned

- **Response:** Thank you for this suggestion. The measurement of the five domains of care-friendliness including equity, accessibility, acceptability, appropriateness, and effectiveness prepared in a separate document and uploaded as supplementary material (to manage the space in the methods part).

14. **Reviewer 1 comment**: There was a problem of organizing your manuscript with the appropriate sequence. For example, sample size determination and sampling procedure must be organized before the data collection procedure. Revise it.

- **Response:** Thank you for your suggestion. Following your and the academic editor's suggestions, we reorganized the methods section (please see the complete response provided under the academic editor's comment 2 above.)

15. **Reviewer 1 comment**: You haven’t used supervisors? Why? What method was used to control data quality?

- **Response:** Thank you for raising this question. We have supervisors as members of the research team for the study. We have added a data quality control section under the methods part. The number, the qualifications, and the role of data collectors and supervisors are explained.

16. **Reviewer 1 comment**: Have you checked the multi-collinearity of independent variables? Have you checked model fitness?

- **Response:** You have raised an important question. We did not check this statistically/ using tests. However, we had to observe the variables to check whether there was such suspicion. None of the variables are suspected of that. In addition, the regression analysis supports our claim, by not resulting in estimates with wider CIs and or larger ORs. Regarding the model fitness, we have run the final model in Stata for HL and checked the model fitness.

17. **Reviewer 1 comment**: The recommendation must be specific and must address significant variables. But your recommendation was too general. See page 27 line 481-486.

- **Response:** Thank you for this suggestion. We have changed the recommendation part as per the suggestion. We also believe that equity and appropriateness that were mentioned in the recommendation part are general and more specific characteristics under equity and appropriateness are identified and incorporated in the document

**REVIEWER 2 COMMENTS:**

18. **Reviewer 2 general comments:** The manuscript assessed friendliness and satisfaction level and their factors through a cross sectional study. Although quality and satisfaction of AYHS could be addressed under many qualitative and quantitative studies of “utilization of AYHS, the problem is still more contextual and may provide additional information to scientific and policy makers. But, the paper may benefit from revising the following comments.

- **Response:** Thank you very much for reading our work, contributing informative comments, and all of your other valuable feedback.

19. **Reviewer 2 comments [Background]:** Your justification “Even though 89 AFHS has been providing care in Ethiopia for nearly 20 years, there is a shortage of scientific research on the level of care that meets international standards” need to be more argued. Because there are many quantitative and qualitative why adolescents were not utilizing the AYFS, indirectly the issue of quality and satisfaction. What would this study makes a difference over those studies?

- **Response:** Thank you for making a fair assessment of this part. The existing literature focuses mainly on examining factors that affect AYFH services utilization and examining quality of care concentrating only on a particular aspect. Therefore, this study provides local, context-specific evidence about the degree of friendliness provided by adolescent and youth health services in Ethiopia by using the WHO Quality Assessment Guidebook

20. **Reviewer 2 comments [Methods]:** How 50% of adolescents are assumed to be satisfied where there are many studies exploring many challenges why adolescents were not using the service. Could it be fair to assume there is no study while there are many studies? And, dependent variables are two in this study, “friendliness” and “satisfaction level”. Did you have any other justification why you did calculate separate sample size and compare the two to select the larger sample size?

- **Response:** Thank you for raising this question: We believe that this could be a limitation of the study. However, during the proposal and data collection stage, we couldn't find similar studies done in similar settings. In addition, assuming 50% would give us a larger sample size at a 5% margin of error relative to other prevalence. So, the current sample size covers any prevalence at the 5% margin of error. Having separate sample sizes is not okay while we assume 50%, it is corrected in the manuscript.

21. **Reviewer 2 comments [Methods]:** You selected health facilities first, then systematically selected adolescents and the study is also facility-based, therefore, there could be a larger variance. Why you were not considering other sample size determination techniques like design effect to increase your sample?

- **Response:** Thank you for raising this question:

1. We selected the health facilities using the lottery method, assuming that they are homogeneous.

2. The sampling design is not that much complex.

3. Time feasibility, based on client flow it would take longer than required/feasible time to get larger samples/ than we considered.

4. Based on the rule of thumbs the sample size is more than enough to make an inference relative to the number of variables considered in the regression.

Considering all of the above we did not consider the design effect.

22. **Reviewer 2 comments [Methods]:** Could the study be generalizable? Because usually most awarded adolescents are usually come frequently and they could have adequate knowledge about the characteristics of your interest. But, majority may not! Don’t you think that this is your limitation of the study?

- **Response:** You have raised an important question. We agree with you, it can be one of the limitations of the study. It is already presented in the document as follows: The use of exit interviews as sources of client satisfaction has limitations in that they are conducted with a sample of clients who have already decided to visit a particular healthcare facility and are likely under the assumption that it will be at least satisfactory.

23. **Reviewer 2 comments [Methods]:** In your analysis, is it fair all the items in each construct could have reasonable factor loading despite easy to assume that they have reasonable reliability coefficients?

- **Response:** Thank you for raising this question. We have adopted the tool from the WHO and assumed that it is a validated tool and standard guide, which is why we did not check the factor loadings using CFA.

24. **Reviewer 2 comments [Methods]:** Many adolescents and youths aged less than 18 years utilize AYFH services without consulting their parents/guardians and asking for parental consent might cause emotional harm to adolescents and youths. Due to this, parents’ consent was not sought, and it was waived by the IRB of SPHMMC.” Could the IRB of SPHMMC waive anyone’s harm or benefit or autonomous? Parents should have been convinced or otherwise it could have been better excluded those under eight adolescents than justifying this way?

- **Response:** Thank you for raising this question: Ethics is always in support of benefits and waiving is also the mandate of the ethics committee for the benefit of the study participants. In this scenario, waiving the consent for the minors outweighs the risk of excluding youth/letting them know their parents. As the problem still exists and excluding minors will cost a huge information gap, waiving was considered as the best option, considering the risks and benefits of waiving the consent form.

25. **Reviewer 2 comments [Results]:** The subheading “Demographic and socio-economic background of study participants” could have been shortened.

- **Response:** Thank you for your suggestion. This is shorted as socio-demographic characteristics.

26. **Reviewer 2 comments [Results]:** In Table 5, how one could know how many variables were taken to the multivariable logistic regression, and which of those are not associated?

- **Response:** Thank you for this suggestion. This part is modified, the number of variables that had a statistical association in the bivariate analysis are presented in the document and the number of variables included in the multivariate logistic regression is also described.

Again, thank you for allowing us to strengthen our manuscript with your valuable comments and queries. We have worked hard to incorporate your feedback and hope that these revisions persuade you to accept our submission.

Sincerely,

Andamlak Gizaw Alamdo

Corresponding Author

Department of Health Service Management, Health Promotion, Reproductive Health, and Nutrition, School of Public Health, Saint Paul's Hospital Millennium Medical College, Addis Ababa, Ethiopia

[gizandal@gmail.com/andamlak.gizaw@sphmmc.edu.et](mailto:gizandal@gmail.com/andamlak.gizaw@sphmmc.edu.et)

+251912038993
