# Supplementary material for: Youth-friendly health service in Ethiopia: Assessment of care friendliness and user’s satisfaction
Source: PLoS One. 2024 Jul 16;19(7):e0307142. doi: 10.1371/journal.pone.0307142 (PMC11251592; doi:10.1371/journal.pone.0307142)
Supplement: Attachment — Submitted filename: Response to Reviewers.docx [file pone.0307142.s005.docx]

# **Rebuttal Letter_v2**

Academic Editor

PLOS ONE

Date: June 27, 2024

**Author’s response to reviews: Version _2**

**Title:** Youth-friendly health service in Ethiopia: Assessment of care friendliness and user’s satisfaction

**Academic editor’s comments**

Many Thanks. We have learnt from your comments, and have tried our best to address them.

**Academic editor’s comment [Abstract]:** Is AOR be used to declare the presence of statistically significant associations? Rewrite this description.

- **Response:** Thank you for pointing this out. We have corrected it.

**Academic editor’s comment [Methods]:** Use the most updated/recent population size of the study areas. Currently, Addis Ababa’s population is more than 5 million.

- **Response:** We noticed the oversight and have corrected it. We have come in more recent data, and have updated the population size accordingly.

1. **Academic editor’s comment [Methods]:** The assumption of including this zone, over other zones/districts all over the country is no sufficiently justified. Strongly recommend to briefly put the explanations of including this district, and no other districts with the service (AYFHS).

**Response:** We appreciate your point. The criteria considered when choosing the East Shewa zone for the study were briefly described again.

2. **Academic editor’s comment [Methods]:** Cite the literature reviewed.

- **Response:** Thank you. We have cited the references reviewed.

3. **Academic editor’s comment [Methods]:** Is this the questionnaire? Use correct labels while supplementary materials.

- **Response:** Yes, it’s the questionnaire. We have revised the label for supplementary material

4. **Academic editor’s comment [Methods]:** Merge this with the operational definitions section. You can name as: **Study variables**

- **Response:** Thank you for your suggestion. We have merged the study variables and operational definition as ‘Study variables”

5. **Academic editor’s comment [Methods]:** OR is measure of strength of association, it is only p-value and 95% CI that is used to declare the presence of statistically significant associations. Correct descriptions accordingly

- **Response:** Thank you, we noticed the error. We revised it accordingly.

6. **Academic editor’s comment [Methods]:** Indicate the date as well under the ethical consideration sub-section.

- **Response:** Thank you. We have incorporated the date and revised a paragraph.

7. **Academic editor’s comment [Results, Table 1]:** The way age variable presented is not clearer, suggest using categories. It is a district, not Oromia. Correct accordingly. Are both outputs similar? Use mean with SD. Suggest categorizing age, and describe the mean results in narrations.

**Response:** Thank you for this suggestion. We have revised this part accordingly. We categorized the age, presented it in the table, and omitted the mean and median results already narrated in the report.

8. **Academic editor’s comment [Results, Table 2-3]:** Describe all acronyms/abbreviations used as table footnote. Try to concise variable names. See highlighted ones and correct. You can describe the details in narration.

**Response:** Revised based on the suggestions. Variable names are revised and the mean and median results are deleted from the table.

9. **Academic editor’s comment [Results, Table 5]:** It would have been good if the reference group be reversed (Factors associated with adolescent and youth client satisfaction).

**Response:** Thank you for raising this concern and we agree with you. It could have been better if the reference category had a larger frequency. However, during data analysis, we have considered other issues such as consistency with previous research, and interpretability (to have a meaningful comparison).

10. **Academic editor’s comment [Results, Table 5]:** What does this (‘1’) represent? Describe as table footnote. Describe all other abbreviations used in the table.

- **Response:** Thank you. "1’: indicates the reference and the baseline category for the binary and multivariable logistic regression analysis, respectively. All abbreviations/acronyms are described as footnotes.

1. **Reviewer 1 Comments:** All comments were addressed adequately. this manuscript is suitable for publication in PLoS One journal fulfilling all criteria.

- **Response:** Thank you. Your input has been very useful in enhancing the paper.

2. **Reviewer 1 and Reviewer 2: Overall comments**

**Response:** Many thanks for your support in enhancing the quality of our paper.

**Final comment:**

**Journal Requirements:** Please review your reference list to ensure that it is complete and correct.

**Response:** Thank you. We have reviewed our reference list and revised it thoroughly according to the journal requirements.

Again, thank you for allowing us to strengthen our manuscript with your valuable comments and queries. We have worked hard to incorporate your feedback

Sincerely,

Andamlak Gizaw Alamdo

Corresponding Author

Department of Health Service Management, Health Promotion, Reproductive Health, and Nutrition, School of Public Health, Saint Paul's Hospital Millennium Medical College, Addis Ababa, Ethiopia

[gizandal@gmail.com/andamlak.gizaw@sphmmc.edu.et](mailto:gizandal@gmail.com/andamlak.gizaw@sphmmc.edu.et)

+251912038993
